# Prognostic markers in Chronic Lymphocytic Leukaemia - A flow cytometric analysis

**DOI:** 10.12669/pjms.36.3.541

**Published:** 2020

**Authors:** Hala Haq, Nasir Uddin, Saleem Ahmed Khan, Sunia Ghaffar

**Affiliations:** 1 Dr. Hala Haq, MBBS, M.Phil (Hematology). Demonstrator Pathology, Fazaia Medical College, Islamabad, Pakistan; 2 Col. Nasir Uddin, MBBS, FCPS. Assistant Professor Pathology, Army Medical College, Rawalpindi, Pakistan; 3 Maj. Gen. Saleem Ahmed Khan, HI (M), MBBS, MCPS, FCPS, FRCP, PhD. Professor Pathology and Principal, Army Medical College, Rawalpindi, Pakistan; 4 Dr. Sunia Ghaffar, MBBS, M. Phil. Army Medical College, Rawalpindi, Pakistan

**Keywords:** Chronic Lymphocytic Leukaemia, Prognostic markers, Immunophenotyping, CD39, ZAP-70, CD49d

## Abstract

**Objective:**

To find out the frequency of ZAP-70, CD38 and CD49d in patients diagnosed with CLL in our population.

**Methods:**

This is a cross sectional study conducted in Army Medical College in collaboration with Armed Forces Institute of Pathology and Military Hospital Rawalpindi from 1^st^ January 2018 to 30^th^ November 2018. Permission from Institutional Ethical Committee was obtained. Blood samples were collected by non-probability consecutive sampling technique and analyzed for blood counts and flow cytometry was done for ZAP-70, CD38 and CD49d. Manufacturer’s instructions for the kits were strictly followed.

**Results:**

Fifty-one newly diagnosed patients with CLL were studied for the prognostic markers in CLL. CD 38 was expressed in 25(49%) and CD49d in 21(41.2%). ZAP-70 expression was not detected in our series of patients.

**Conclusion:**

We conclude that CD38 and CD49d expression was detected in almost half of the patients of CLL in our series. CD49d showed statistically positive correlation with CD38, showing that it is a more pragmatic choice for reliable prognostication of CLL along with CD38.

## INTRODUCTION

Chronic Lymphocytic Leukemia (CLL) is characterized by accumulation of small mature looking, ineffectual, CD5+ B-cells in the peripheral blood, bone marrow and secondary lymphoid tissues.^1^ These cells have a characteristic immunophenotype i.e. CD19+, CD20+ and CD23+with relatively low expression of CD22 and CD79b.^2^

The peak incidence of CLL is between 60 to 80 years, and only 10% of patients are younger than 55 years of age. In Pakistan it accounts for 20.1% of all leukemias.^3^ It is more common in the West with an incidence of 4.2:100,000 per year.^3^ Most cases are diagnosed during routine complete blood counts done for other reasons.^4^

Its diagnosis is based upon clinical presentation and laboratory features.^5^ Peripheral blood counts with absolute lymphocyte count more than 5 x 10^9^/L, which may reach to 300 x 10^9^/L. The peripheral smear shows small mature looking lymphocytes with numerous smudge cells. A bone marrow biopsy, if done, shows predominance of mature lymphocytes, replacing 95% of normal hemopoietic tissue. Immunophenotyping is done to confirm the diagnosis.^6^

The Rai and Binet staging systems are well recognized systems which serve as standard for assessing treatment requirements and overall survival in the patients diagnosed with CLL. However, both these systems are unable to categorize the patients into groups requiring treatment from those in whom the disease remains indolent.^7^ The outcome of patients can be predicted by a number of immunophenotypic markers (ZAP-70, CD38 and CD49d), Immunoglobin heavy variable (IGHV) gene mutation and chemical analysis.^8^

ZAP-70 and CD38 are well-established immunophenotypic markers indicating poor prognosis in CLL. ZAP-70 is a protein rarely present on normal B-lymphocytes whereas CD38 is expressed on them. Patients who are positive for ZAP-70 and CD38 have a poor prognosis with aggressive disease course and a shorter overall survival. On the other hand those who are negative for ZAP-70 and CD38 have much better results.^7^,^9^

CD49d is a recently added prognostic marker. It belongs to the integrin family with an important function in leukocyte activation, trafficking and survival.^10^ Its expression promotes unfavorable progressive disease course and can identify patients with poor disease outcome, independent of CD38 and ZAP-70.^8^ Studies show that CD49d positive patients have increased risk of death and lower overall survival, independent of ZAP-70 and CD38. Comparing models of these three prognostic markers, with and without CD49d by several prediction performance measures indicated that excluding CD49d significantly reduced the prognostic power of the model.^11^ Another study compared the combined expression of CD38 and CD49d. They showed that all those patients who require treatment had strong double expression of both. On the contrary, those with double negative expression had good prognosis and long treatment free survival.^12^ Patients with > 30% of CLL B-cell expressing CD49d are labeled positive.^11^ Till to date no study has been done in Pakistan to study presence and frequency of CD49d in Pakistani patients with CLL.

This study helps us to evaluate the prognosis of CLL at the time of diagnosis in our population. ZAP-70, CD38 and CD49d are prognostic markers of CLL. They help in segregating those patients of CLL which will need treatment from those that can be placed in the “Wait and watch” group. Globally, studies show that CD49d is gaining acceptance as an independent prognostic marker and may replace the combination of ZAP-70 and CD38. Foregoing in view, we planned a study to find out the frequency of ZAP-70, CD38 and CD49d in CLL patients our setup.

## METHODS

This cross sectional study was conducted in Department of Hematology, Army Medical College in collaboration with Armed Forces Institute of Pathology Rawalpindi from 1^st^ January, 2018 to 31^st^ October, 2018 after the approval of Institutional Review Board dated on January 24, 2019.

Total 51 newly diagnosed cases of CLL were included in our study. Sample size (n=44) was calculated by WHO calculator (Confidence interval at 95% and Anticipated population as 13% with absolute precision required as 5%). Sample collection was done by non-probability purposive sampling.

Three (3) ml of venous blood was drawn under aseptic conditions. It was transferred to Ethylenediaminetetraacetic acid (EDTA) tube. Complete blood counts were generated through Sysmex KX-21TM automated hematology analyzer after adequate quality control. Immunophenotyping was performed by flow cytometry to analyze for ZAP-70, CD38 and CD49d by BD FACS caliber and BD FACS CANTO. Known negative samples were used as normal controls. Manufacturer’s instructions for the kits used were strictly followed.

### Statistical analysis

Data was analyzed by statistical package for social sciences (SPSS 23). For qualitative variables frequency and percentages were calculated and quantitative variables Mean and Standard Deviation (SD) were calculated. Correlation between CD38 and CD49d was calculated by applying the Pearson Chi-Square. P-value <0.05 was considered statistically significant.

## RESULTS

Our study included a total of 51 newly diagnosed cases of CLL. Out of these, 40 (78%) were male and 11 (21.5%) were females. The mean age of the patients was 65 years± 10.64 (mean ± SD) years with a range of 39 – 86 years. The mean hemoglobin, white cell and platelets counts were 11.1 ± 2.7 g/dl (range 3.6 – 16.1); 80.8 ± 74.2 x 10^9^/L (range 11.7 – 538x10^9^/L) and 169 ± 67 x 10^9^/L(range 08 – 383x10^9^/L) respectively. The mean absolute lymphocyte count was 68.2 ± 71.6 x 10^9^ / L (range 7.9 – 505.7 x 10^9^/L) (Table-I).

**Table-I:** Mean and standard deviation of quantitative variables.

	Minimum	Maximum	Mean	Std. Deviation
Age	39	86	65.8	10.64
Total Leucocytes Count	11.7	538	80.87	74.2
Absolute Lymphocyte Count	7.9	505.7	68.2	71.6
Hemoglobin	3.6	16.1	11.1	2.7
Lymphocyte Count	45	99	80.6	11.3
Platelet Count	8.0	383	169.7	67.4

**Fig.1:**
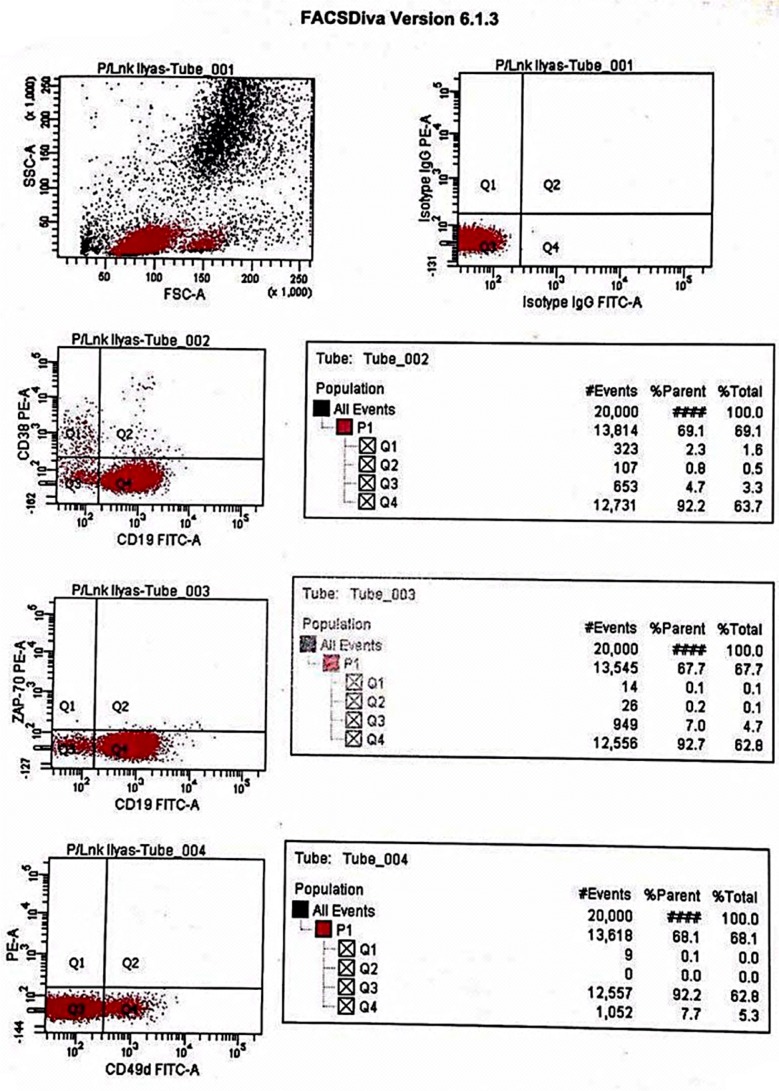
Dot-plot for Zap-70, CD38 and CD49d.

CD38 expression was detected in 25 (49%) cases with the mean value of 34.4% (range 0-96) while CD49d was expressed in 21 (41.2%) cases with a mean of 35.4 (range 0-99). ZAP-70 expression was not detected in any case (Fig.1). The mean of positive expression of CD38 was 70.9 ± 18.2 with range of 42%-96% and CD49d was 76.2 ± 16.2 with a range of 42%-99% (Table-II).

**Table-II:** Mean and range of the CD49d and CD38.

Marker expression (no)		Mean ± SD	Range %
*CD49d	Positive (21)	76.2 ± 16.1	42%-99%
	Negative (30)	6.9 ± 7.1	0%-26.8%
**CD38	Positive (25)	70.9 ± 18.2	42%-96%
	Negative (26)	2.1 ± 3.9	0%-13%

* CD49d ≥30% is considered positive,

** CD38 ≥30% is considered positive.

**Fig.2:**
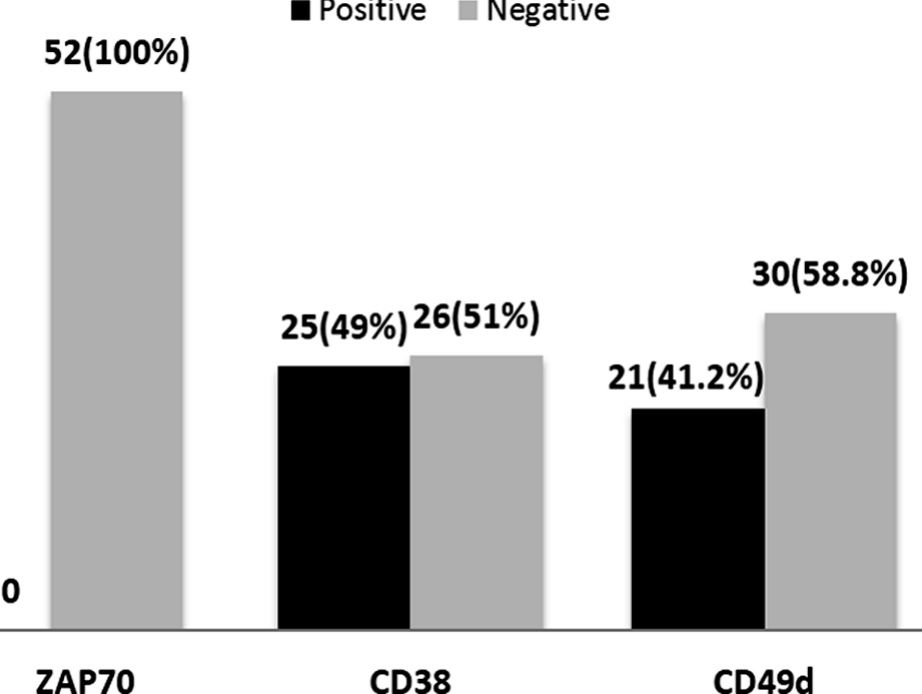
Frequency of ZAP-70, CD38 and CD49d.

We calculated the correlation between CD38 and CD49d by applying the Pearson Chi-Square. The p-value was <0.05 showing that the two variables are statistically significant (Fig.2). We divided our data into two groups i.e. Group-1 (below 60 years of age) and Group-2 (61 and above). In Group-1, 3/13 cases were positive for CD49d and CD38. In Group-2, 18/38 cases were positive for both CD38 and CD49d. No positive association was found between CD markers and age group (p-value >0.05).

## DISCUSSION

Chronic lymphocytic leukemia is the most frequently occurring chronic leukemia in the Western world.^7^ The progression and response to treatment is variable. Previously, prognosis of patients with CLL was based on the clinical features alone. However with the advent of new techniques, significant progress has been seen like identification of immunophenotypic markers and molecular genetics which help us to predict the progression of the disease.^13^,^14^

We studied 51 newly diagnosed patients of CLL. Among these, 25 (49%) were positive for CD38.Our study is consistent with other studies conducted in China, India and Iraq showing almost similar results i.e. CD38 positivity was found in 56.7%,44% and 30.6% of patients.^15^-^17^ However, our values are slightly higher than those reported by Wiestner A et al. in UK and D’Arena G et al. in Italy which were 30% and 29% respectively.^18^,^19^

CD49d was positive in 21 (41.2%) cases which is comparable to results by Bulian P et al. in Italy, Uzay A et al in Turkey and Gattei V et al. in Italy who reported CD49d positivity in 52%, 47% and 39% of cases respectively.^11^,^20^,^21^ Our results were slightly lower than study done by Al-Rubaie HA et al.^15^ in Iraq who showed positivity for CD49d (60%). This might be explained by the smaller sample size they studied (n=30). We could not find any similar studies conducted in our region with which our statistics could be compared. So we believe that this is the first study of this kind in Pakistan.

The expression of ZAP-70 was not recorded in our cases. A study conducted by Zeeshan R et al. showed ZAP-70 expression in only 13% cases.^7^ On the contrary, an Indian study revealed that expression of ZAP-70 by flow cytometry was weak in a vast majority of cases (n=60) with small shifts above the baseline threshold thus, was not a robust assay. This may explain why ZAP-70 could not be detected in any of our studied cases. And hence low frequency in South Asian population.^22^

There was a significant correlation between CD38 and CD49d (p-value <0.05). No correlation was found between ZAP-70 and either of the other two markers (CD38 & CD49d). We divided our data into two groups i.e. Group-1 (below 60 years of age) and Group-2 (60 and above). No association was found between the immunophenotypic markers and age.

In an analysis of 3000 patients done in Italy for flow cytometric based prediction of overall survival (OS) in CLL, CD49d+ had a significantly high risk of death and lower poor survival rate as compared to CD49d- patients. By a Cox analysis for OS, they showed that CD49d+ patients have a two-fold increase risk of death and it was the only flow cytometry based marker with independent prognostic relevance for OS. They also compared models for prognostic markers with and without CD49d by several prediction performance measures indicating that excluding CD49d significantly reduced the prognostic power of the model.^11^

In 2017, a study conducted by Ahmed S et al.^12^ compared the expression of CD38 and CD49d in patients with CLL. They concluded that patients with strong double expression of CD38 and CD49d required treatment. Whereas, patients with negative expression of CD38/CD49d had a good prognosis and long treatment free survival.

### Limitation of the study

The limitation in our study was that only a small number of patients were studied. It is strongly recommended that all the three prognostic markers i.e. CD38, ZAP-70 and CD49d are studied in all the newly diagnosed patients of CLL. As the study was single centered, that’s why it’s difficult to extrapolate it to whole Pakistani population. Therefore, it is highly recommended that these immunophenotypic markers should be studied on a large cohort of patients in centers which extend diagnostic and therapeutic facilities to patients of Chronic Lymphocytic Leukemia in Pakistan. Thus, this pilot study can form a foundation for larger studies on the subject.

## CONCLUSION

We conclude that assessing prognosis of CLL at the time of diagnosis is essential for all patients to segregate patients into groups who need urgent treatment because of presence of adverse prognostic markers from those who can be placed in watch and wait group. CD38 and CD49d expression was detected in almost half of the patients and were significantly correlated. CD49 is gaining acceptance internationally as an independent prognostic marker and is more reliable for prognostication of CLL along with CD38.

### Recommendation

We recommend that along with the conventionally used biomarkers i.e. ZAP-70 and CD38, CD49d should also be added to the immunophenotyping panel for stratification of prognostic groups in CLL. Further studies should also be done on larger groups of patients to evaluate the frequency of these biomarkers in our population and re-evaluation should be done for the inclusion of ZAP-70 in the immunophenotyping panel.

### Authors’ Contribution:

**HH** conceived, designed and did statistical analysis & manuscript writing.

**NUD** did overall supervision, proof reading and final drafting.

**SAK** did the conceptualization of the study and critical revision of article.

**HH, SG** did data collection and statistical analysis.

**HH** takes the responsibility and is accountable for all aspects of the work in ensuring that questions related to the accuracy or integrity of any part of the work are appropriately investigated and resolved.
